# Time series of freshwater macroinvertebrate abundances and site characteristics of European streams and rivers

**DOI:** 10.1038/s41597-024-03445-3

**Published:** 2024-06-07

**Authors:** Ellen A. R. Welti, Diana E. Bowler, James S. Sinclair, Florian Altermatt, Mario Álvarez-Cabria, Giuseppe Amatulli, David G. Angeler, Gaït Archambaud, Iñaki Arrate Jorrín, Thomas Aspin, Iker Azpiroz, Nathan Jay Baker, Iñaki Bañares, José Barquín Ortiz, Christian L. Bodin, Luca Bonacina, Núria Bonada, Roberta Bottarin, Miguel Cañedo-Argüelles, Zoltán Csabai, Thibault Datry, Elvira de Eyto, Alain Dohet, Sami Domisch, Gerald Dörflinger, Emma Drohan, Knut A. Eikland, Judy England, Tor E. Eriksen, Vesela Evtimova, Maria J. Feio, Martial Ferréol, Mathieu Floury, Maxence Forcellini, Marie Anne Eurie Forio, Riccardo Fornaroli, Nikolai Friberg, Jean-François Fruget, Jaime R. Garcia Marquez, Galia Georgieva, Peter Goethals, Manuel A. S. Graça, Andy House, Kaisa-Leena Huttunen, Thomas Correll Jensen, Richard K. Johnson, J. Iwan Jones, Jens Kiesel, Aitor Larrañaga, Patrick Leitner, Lionel L’Hoste, Marie-Hélène Lizée, Armin W. Lorenz, Anthony Maire, Jesús Alberto Manzanos Arnaiz, Brendan Mckie, Andrés Millán, Timo Muotka, John F. Murphy, Davis Ozolins, Riku Paavola, Petr Paril, Francisco Jesús Peñas Silva, Marek Polasek, Jes Rasmussen, Manu Rubio, David Sánchez Fernández, Leonard Sandin, Ralf B. Schäfer, Astrid Schmidt-Kloiber, Alberto Scotti, Longzhu Q. Shen, Agnija Skuja, Stefan Stoll, Michal Straka, Rachel Stubbington, Henn Timm, Violeta G. Tyufekchieva, Iakovos Tziortzis, Yordan Uzunov, Gea H. van der Lee, Rudy Vannevel, Emilia Varadinova, Gábor Várbíró, Gaute Velle, Piet F. M. Verdonschot, Ralf C. M. Verdonschot, Yanka Vidinova, Peter Wiberg-Larsen, Peter Haase

**Affiliations:** 1 Department of River Ecology and Conservation, Senckenberg Research Institute and Natural History Museum Frankfurt, Gelnhausen, 63571 Germany; 2 Conservation Ecology Center, Smithsonian National Zoo and Conservation Biology Institute, Front Royal, Virginia 22630 USA; 3 Department of Ecosystem Services, German Centre for Integrative Biodiversity Research (iDiv) Halle-Jena-Leipzig, Leipzig, 04103 Germany; 4 Institute of Biodiversity, Friedrich Schiller University Jena, Jena, 07743 Germany; 5 Department of Ecosystem Services, Helmholtz Center for Environmental Research - UFZ, Leipzig, 04318 Germany; 6 Department of Evolutionary Biology and Environmental Studies, University of Zurich, 8057 Zürich, Switzerland; 7 Department of Aquatic Ecology, Eawag: Swiss Federal Institute of Aquatic Science and Technology, 8600 Dübendorf, Switzerland; 8 IHCantabria - Instituto de Hidráulica Ambiental de la Universidad de Cantabria, Santander, 39011 Spain; 9 School of the Environment, Yale University, New Haven, CT 06511 USA; 10 Department of Aquatic Sciences and Assessment, Swedish University of Agricultural Sciences, Uppsala, 75651 Sweden; 11 IMPACT, the Institute for Mental and Physical Health and Clinical Translation, Deakin University, Geelong, Victoria Australia; 12 Brain Capital Alliance, San Francisco, CA USA; 13 School of Natural Resources, University of Nebraska-Lincoln, Lincoln, NE USA; 14 INRAE, Aix Marseille Univ, RECOVER, Aix-en-Provence, 13182 France; 15 Agencia Vasca del Agua, Vitoria-Gasteiz, 01013 Spain; 16 Environment Agency, Bristol, UK; 17 Ekolur Asesoría Ambiental SLL, Oiartzun, 20180 Spain; 18 Laboratory of Evolutionary Ecology of Hydrobionts, Nature Research Centre, Akademijos Str. 2, Vilnius, 08412 Lithuania; 19 Departamento de Medio Ambiente y Obras Hidráulicas, Diputación Foral de Gipuzkoa, Donostia-San Sebastián, 20004 Spain; 20 LFI - The Laboratory for Freshwater Ecology and Inland Fisheries, NORCE Norwegian Research Centre, Bergen, 5838 Norway; 21 Department of Earth and Environmental Sciences - DISAT, University of Milano-Bicocca, Milan, 20126 Italy; 22 FEHM-Lab (Freshwater Ecology, Hydrology and Management), Department of Evolutionary Biology, Ecology and Environmental Sciences, Facultat de Biologia, Institut de Recerca de la Biodiversitat (IRBio), University of Barcelona, Barcelona, 08028 Spain; 23 Eurac Research, Institute for Alpine Environment, Bolzano/Bozen, 39100 Italy; 24 FEHM-Lab, Institute of Environmental Assessment and Water Research (IDAEA), CSIC, Carrer de Jordi Girona, 18-26, 08034 Barcelona, Spain; 25 HUN-REN Balaton Limnological Research Institute, 3 Klebelsberg Kuno, H8237 Tihany, Hungary; 26 Department of Hydrobiology, University of Pécs, Pécs, 7624 Hungary; 27 INRAE, UR RiverLy, Centre de Lyon-Villeurbanne, Villeurbanne, F-69625 France; 28 Fisheries Ecosystems Advisory Services, Marine Institute, Newport, F28PF65 Ireland; 29 Environmental Research and Innovation department, Luxembourg Institute of Science and Technology, Esch-sur-Alzette, L-4362 Luxembourg; 30 Department Community and Ecosystem Ecology, Leibniz Institute of Freshwater Ecology and Inland Fisheries (IGB), Berlin, 12587 Germany; 31 Water Development Department, Ministry of Agriculture, Rural Development and Environment, Nicosia, 1047 Cyprus; 32 Centre for Freshwater and Environmental Studies, Dundalk Institute of Technology, Dundalk, A91 K584 Ireland; 33 Norwegian Institute for Nature Research (NINA), Oslo/Lillehammer, Norway; 34 Norwegian Institute for Water Research (NIVA Denmark), 2300 Copenhagen S, Denmark; 35 Department of Aquatic Ecosystems, Institute of Biodiversity and Ecosystem Research, Bulgarian Academy of Sciences, Sofia, 1000 Bulgaria; 36 Department of Life Sciences, University of Coimbra, Marine and Environmental Sciences Centre, ARNET, Coimbra, 3000-456 Portugal; 37 University of Lyon, Université Claude Bernard Lyon 1, CNRS, ENTPE, UMR 5023 LEHNA, F-69622 Villeurbanne, France; 38 Department of Animal Sciences and Aquatic Ecology, Ghent University, Ghent, 9000 Belgium; 39 University of Copenhagen, Freshwater Biological section, 2100 Copenhagen, Denmark; 40 University of Leeds, water@leeds, School of Geography, Leeds, UK; 41 ARALEP - Ecologie des Eaux Douces, Villeurbanne, 69603 France; 42 Department of Ecology and Genetics, University of Oulu, Oulu, 90014 Finland; 43 Nature Solutions Unit, Finnish Environment Institute, Oulu, 90014 Finland; 44 School of Biological and Behavioural Sciences, Queen Mary University of London, London, E1 4NS UK; 45 Department of Hydrology and Water Resources Management, Christian-Albrechts-University Kiel, Institute for Natural Resource Conservation, Kiel, 24118 Germany; 46 Department of Plant Biology and Ecology, University of the Basque Country, Leioa, 48940 Spain; 47 Department of Water, Atmosphere and Environment, Institute of Hydrobiology and Aquatic Ecosystem Management, University of Natural Resources and Life Sciences, Vienna, Austria; 48 Department of Water, Atmosphere and Environment, Institute of Hydrobiology and Aquatic Ecosystem Management, 1180 Vienna, Austria; 49 Faculty of Biology, University of Duisburg-Essen, Essen, 45141 Germany; 50 EDF Recherche et Développement, Laboratoire National d’Hydraulique et Environnement, Chatou, 78401 France; 51 Department of Ecology and Hydrology, University of Murcia, Murcia, 30100 Spain; 52 Institute of Biology, University of Latvia, Riga, LV-1004 Latvia; 53 Oulanka Research Station, University of Oulu Infrastructure Platform, Kuusamo, 93900 Finland; 54 Water, Energy and Environmental Engineering Research Unit, Faculty of Technology, University of Oulu, 90014 Oulu, Finland; 55 Department of Botany and Zoology, Faculty of Science, Masaryk University, Brno, 61137 Czech Republic; 56 Research Center One Health Ruhr, University Alliance Ruhr, Universitätsstrasse 2, 45141 Essen, Germany; 57 APEM Ltd, Riverview, A17 - The Embankment Business Park – SK4 3GN, Heaton Mersey, Stockport, UK; 58 Institute for Green Science, Carnegie Mellon University, Pittsburgh, 15213 USA; 59 Department of Environmental Planning and Technology, University of Applied Sciences Trier, Birkenfeld, 55761 Germany; 60 T.G. Masaryk Water Research Institute, p.r.i., Brno, 61200 Czech Republic; 61 School of Science and Technology, Nottingham Trent University, Nottingham, NG11 8NS UK; 62 Estonian University of Life Sciences, Chair of Hydrobiology and Fishery, Centre for Limnology, Elva vald, 61117 Estonia; 63 Wageningen Environmental Research, Wageningen University and Research, Wageningen, 6708 PB Netherlands; 64 Flanders Environment Agency, Aalst, 9300 Belgium; 65 South-West University “Neofit Rilski”, Faculty of Mathematics and Natural Sciences, Department of Geography, Ecology and Environment Protection, Blagoevgrad, Bulgaria; 66 Department of Tisza River Research, HUN-REN Centre for Ecological Research, Institute of Aquatic Ecology, Debrecen, 4026 Hungary; 67 Department of Biological Sciences, University of Bergen, Bergen, 5006 Norway; 68 Institute for Biodiversity and Ecosystem Dynamics, University of Amsterdam, Amsterdam, 1098 XH Netherlands; 69 Department of Ecoscience, Aarhus University, 8000 Aarhus C, Denmark

**Keywords:** Freshwater ecology, Community ecology, Biodiversity

## Abstract

Freshwater macroinvertebrates are a diverse group and play key ecological roles, including accelerating nutrient cycling, filtering water, controlling primary producers, and providing food for predators. Their differences in tolerances and short generation times manifest in rapid community responses to change. Macroinvertebrate community composition is an indicator of water quality. In Europe, efforts to improve water quality following environmental legislation, primarily starting in the 1980s, may have driven a recovery of macroinvertebrate communities. Towards understanding temporal and spatial variation of these organisms, we compiled the TREAM dataset (Time seRies of European freshwAter Macroinvertebrates), consisting of macroinvertebrate community time series from 1,816 river and stream sites (mean length of 19.2 years and 14.9 sampling years) of 22 European countries sampled between 1968 and 2020. In total, the data include >93 million sampled individuals of 2,648 taxa from 959 genera and 212 families. These data can be used to test questions ranging from identifying drivers of the population dynamics of specific taxa to assessing the success of legislative and management restoration efforts.

## Background & Summary

Freshwater ecosystems have been altered by a wide range of human impacts since the onset of the industrialization^1,2^, which have increased in intensity in Europe since World War II^3^. Legislation in Europe and elsewhere (e.g., CWA 1972^4^, WFD 2000^5^) has aimed to restore freshwater and riparian habitats, especially through improvements in wastewater treatment and reductions in airborne pollutants^6^. Other stressors such as climate change, land use change, physical alterations to waterways, including straightening, deepening, and impoundments, micropollutants, and invasive species, have either increased in intensity or continue to threaten freshwater ecosystems^7^. Moreover, despite some improvements, most freshwater ecosystems still fail to meet the European Water Framework Directive’s target of good ecological status^8^. Given freshwater biotas’ high biodiversity, importance for ecosystem functioning and provision of ecosystem services, monitoring the state of freshwater ecosystems remains an important task for managers and scientists.

Freshwater macroinvertebrates are commonly used as indicators of the status of freshwater ecosystems because of their taxon-specific sensitivity to environmental conditions. Long-term trends of freshwater communities have typically been examined at local to national scales^9–11^. To study large-scale patterns in biodiversity change, many efforts have been made to compile data on the abundance and occurrence of species into harmonized datasets (e.g., BioTIME^12^, Living Planet Index^13^, CESTES^14^). However, both freshwater biota and all invertebrates are often underrepresented in datasets of species abundance and occurrences. Currently, datasets focused on freshwater ecosystems include an earlier but smaller dataset of European macroinvertebrates^15^, macroinvertebrate communities in intermittent rivers^16^, abundance time-series data for fish^17^, and occurrence records for selected macroinvertebrate groups^18^. Freshwater insects (which constitute a large proportion of macroinvertebrates) are also represented by the InsectChange dataset^19^, but this dataset targeted community-level aggregated data and only the most publicly accessible time-series. In the United States, freshwater communities have been sampled across many sites by federal agencies, but most sites have been sampled only one to three times in the past three decades^20^. Hence, the TREAM (Time seRies of European freshwAter Macroinvertebrates) dataset, presented here, fills an important gap in data availability.

Here, we provide a new dataset of time-series abundance data for freshwater macroinvertebrates in Europe. We used our collaborator network to identify unpublished datasets and published but inaccessible dataset*s* collected within different studies and in different countries. In total, data were sourced from 82 data co-authors including scientists, site managers, and representatives of regulatory agencies from 22 European countries. The TREAM dataset presented here differs from previously published datasets in its inclusion of longer time series (minimum of eight sampling years), of time series reporting all sampled members of the macroinvertebrate community (e.g. not time series of only Odonata or only Ephemeroptera, Plecoptera, and Trichoptera), and its larger number of time series (1,816) within one taxonomic group. Our dataset is outlined in detail below.

We have recently used our dataset to explore how trends in freshwater macroinvertebrate communities have changed through time^1^. The findings from this study indicate an overall increase in the abundance and richness of freshwater macroinvertebrates in Europe since 1968. However, the recovery has slowed since 2010. We further analysed site-level variation in trends in relation to environmental drivers, such as climate and land use^1^. Our dataset can be used for population-level and community-level analyses that address a wide range of questions about spatio-temporal patterns in taxonomic or functional taxon units^21,22^.

## Methods

### Data call and criteria

**Fig. 1:**
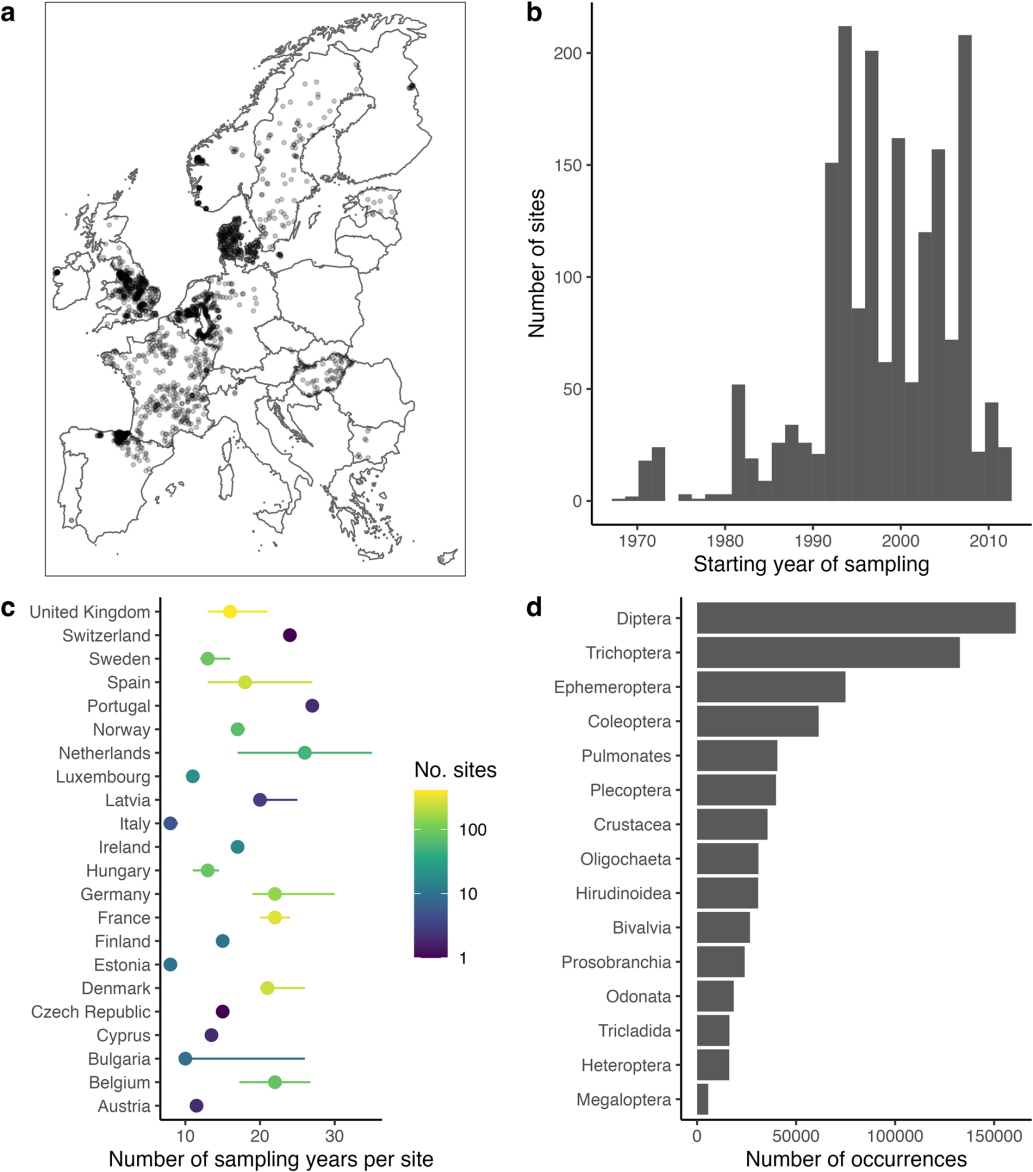
Overview of dataset coverage including the distribution of sampling sites across 22 European countries (**a**), a histogram of the number of sampling sites per a given first year of sampling (**b**), the number of sampling years (median and interquartile range) per site within each country (**c**), and the top 15 most sampled orders and their occurrences across all surveys (**d**).

Data were sourced following a data call to water quality managers and freshwater ecologists in Europe from our professional networks. To be included in the TREAM dataset, data had to meet a number of criteria. First, we only included data that formed a time series (i.e., repeated sampling at the same location), with a minimum of eight sampling years, but sampling years did not have to be consecutive. Second, each time series represents whole sampled macroinvertebrate communities (i.e., all macroinvertebrates sampled were recorded). Third, we included only time series with counts or estimates of taxon abundance at the family, genus, or species level. Fourth, sampling location and method must be consistent over time within each time series, including the sampling method, the sampling effort, and the taxonomic resolution to which individual taxa were identified (see Usage notes regarding taxonomic resolution). Finally, all samples in a time series had to be collected within a period of three consecutive months of the year (e.g., June-August). Data were collected as part of 41 independent monitoring projects; project ID numbers and data provider names are listed in the file “TREAM_siteLevel.csv” within the deposited dataset^23^. Projects reported data from one or more sampling locations per country (Fig. 1; Austria: 2 time series, Belgium: 82, Bulgaria: 9, Cyprus: 2, Czechia: 1, Denmark: 248, Estonia: 10, Finland: 10, France: 307, Germany: 151, Hungary: 87, Ireland: 16, Latvia: 3, Luxembourg: 20, Netherlands: 51, Norway: 67, Portugal: 2, Spain: 245, Sweden: 91, Switzerland: 1, United Kingdom: 406).

### Data standardization

Following the data call, we standardized data by filtering subsets that did not meet our criteria listed above. For example, we excluded samples or whole time series for which sampling methods, effort, or primary level of taxonomic resolution changed during the time series (see Usage notes for a caveat regarding taxonomic resolution). When time series did not meet our inclusion criteria, including consistent sampling methods, for the minimum of eight sampling years, we excluded the whole time series. We only allowed one sampling event per year defined as sampling that occurred within a single day. For time series where multiple samples were provided within the same year, we excluded any sampling outside the most sampled period of three consecutive months. We excluded taxa that are not freshwater invertebrates, e.g., terrestrial taxa, vertebrates, and microinvertebrates that were not present in most of the time series, such as mites and copepods. Taxon names were harmonized according to freshwaterecology.info^24^.

### Data description

The resulting TREAM dataset consists of 1,816 time series from 22 European countries. Time series had a mean of 14.9 (median: 14; range: 8–32) sampling years (not necessarily continuous, spanning 8–39 years). The most commonly sampled families by abundance were Chironomidae, Gammaridae, and Hydrobiidae, and by frequency of occurrence in site sampling years were Chironomidae, Baetidae, and Elmidae. Taxonomically, samples within time series were resolved to family level or finer (762 time series mainly at species level resolution, 537 at genus/mixed level, and 517 at family level). Samples were taken from the riverbed sediments and the majority of collections used forms of kick-net sampling, with either a fixed area or time-based sampling effort. Metadata including information on sampling effort and methods in terms of sampling approach and units are provided in the Knowledge Network for Biocomplexity repository^23^.

### Environmental data

For each sampling location, we included stream characteristics (listed below), climate (cumulative precipitation and mean maximum temperature during the 12-month period before the sampling period), land cover (urban and agricultural land use), and dam impact score. To determine the sampling area of each site to extract these environmental data, we first assigned each sampling site to the corresponding stream segment of the Hydrography90m stream network^25^ using the *v.net* function in GRASS GIS^26^. The longest distance within the stream segment subcatchment was used as the distance threshold for the snapping function. We then computed the upstream catchment of each sampling site using the *r.water.outlet* function and stored the output as a raster GeoTIFF file. This raster file served as the basis for the subsequent environmental data extractions. While the point snapping and catchment delineation procedure were automated, we reviewed each resulting catchment. In cases where the site was assigned to the wrong segment, we manually corrected the stream segment assignment for a given sampling site and repeated the procedure. For stream characteristics, we extracted the topographical and topological attributes of each stream segment of the Hydrography90m dataset^25^. Specifically, we provide the extracted area of each site’s subcatchment, Strahler stream order, flow accumulation, elevation, and slope.

For climate data, we used the TERRA Climate monthly time series from 1967–2020 at 4-km resolution^27^ and overlaid the monthly precipitation and temperature raster layers with the upstream catchment of each sampling site using the *r.univar* function in GRASS GIS. In addition, we extracted the local temperature layers at each sampling site using the *gdallocationinfo* function. This resulted in monthly averaged upstream aggregated precipitation (in mm) and monthly averaged local maximum temperature (in °C). For each site and year, we then calculated the average of the monthly temperatures, and the sum of the monthly precipitation values for the year preceding the average month of sampling (e.g., for a site with most sampling occurring in May, year was defined as the preceding 12 months of May until April). The use of climatic data from one year prior to the start of the first time series allows the capture of the annual period of weather prior to the first sampling event, since population responses are typically lagged. We used Bayesian models in the program R v. 4.2^28^ and package brms^29^ to calculate trends of change in precipitation and temperature over time (all sampling years of a given site/time series). Code for climate trends can be found at: https://github.com/Ewelti/EuroAquaticMacroInverts/blob/main/R/Initial_Biodiversity_FuncTrait_and_climate_calcs/climateTrends_brms.R.

To collect data on land cover, we used the ESA Land Cover CCI Product^30^ at 300-m spatial resolution to extract the percent coverage of the urban and crop land-cover categories from annual maps, available from 1992–2018. We first split each annual multi-category land cover raster layer into the single raster layers that hold only each single category. We then overlaid the urban and crop land cover category raster layers with the subcatchment raster layers and used the *r.univar* function to yield annual cover of the upstream urban and crop land cover within the catchment of each sampling site.

We used the GRanD dataset^31^ to obtain the dams across the study area domain. First, we assigned each dam to the Hydrography90m network using the *v.net* function and using the longest distance within the stream segment subcatchment as the distance threshold. Afterwards, we visually checked the spatial assignment. We then computed the distance from each sampling site to the next upstream dam using the GRASS GIS function *v.net.distance*^25^. To create a proxy for dam impacts, we first extracted only dams within 100 km of a site that were connected to the site and upstream of the site. We then calculated a dam impact score for each site as:

$$\mathop{\sum }\limits_{i=1}^{n}\frac{100-{d}_{i}}{100}$$

where *n* represents the number of dams, *i* denotes a given dam, and *d* is the distance (km) of the dam from a site.

## Data Records

Data are available from the Knowledge Network for Biocomplexity repository (https://knb.ecoinformatics.org/view/doi:10.5063/F1NG4P4R)^23^. Data are provided in three csv files listed below:

### TREAM_siteLevel.csv

This file contains summary TREAM data where each row corresponds to a site (a site = location of one time series). Data include site characteristics (e.g. coordinates, number of sampling years, sampling season), data providers, and primary taxonomic resolution level, summary information on biodiversity across the time series (e.g. total taxa richness for all sampling years). The majority of available corresponding environmental data (i.e., dam impact score, mean values across sampling years of the mean temperature and precipitation in the 12 months prior to sampling, slope of temperature and precipitation over the sampled years, and percent upstream area of cropland and urban cover) and stream characteristic variables (i.e., area of subcatchment, Strahler stream order, flow accumulation, elevation, and slope) are included in this file in addition to sampling method information (e.g., sampling units, primary taxonomic resolution).

### TREAM_siteYEARLevel.csv

This file contains TREAM data where each row corresponds to a site and a year of sampling. This file is the primary source for biodiversity summary metrics (e.g., taxa richness, functional redundancy, abundance of native taxa for each site and year) associated with this dataset. Environmental data that are available on an annual basis (i.e., mean temperature and precipitation in the 12 months prior to sampling, and percent upstream area of cropland and urban cover) are also provided in this file.

### TREAM_alltaxa.csv

This file contains raw biodiversity data where each row corresponds to a taxon within a site and year of sampling. The abundance of each taxon per sampling unit (i.e., all sampling within one site and one time point) is included in this file (see TREAM_siteLevel.csv for sampling methods and effort information).

## Technical Validation

Technical validation of the TREAM dataset was achieved through exclusion of time series data that did not match our inclusion criteria and data standardisation steps (outlined in Methods above). Any noted issues that did not adhere to the outlined standardisation within the datasets from the 41 independent projects included in this dataset were checked with data providers and corrected or removed when standardisation was not achievable (e.g., when collection methods changed over the course of the time series).

## Usage Notes

Several key characteristics about these data should be noted by future data users. First, and most importantly, sampling methods and effort, and seasonality are standardised within individual time series but can vary across time series. This means that raw data are not directly comparable across the 41 independent projects included in the TREAM dataset.

Second, while we use the taxonomic backbone of freshwaterecology.info^23^ as it is a common tool used by European freshwater ecologists, we are aware that it does not capture all recent changes to taxonomic names.

Third, two pairs of time series overlap in sampling locations: 1) Site ID = 100000001 (SVD) & 100000309 (Bugey_SVD) refer to the same location; 2) Site ID = 100000002 (SVG) & 100000308 (Bugey_SVG) refer to the same location. The data from these sites were collected by the Institut national de la recherche agronomique (referent: Maxence Forcellini) between 1980 and 2014, and by Électricité de France (Referent: Anthony Maire) between 2000 and 2019.

Fourth, although standardised taxonomic resolution within time series was a criterion for data inclusion, some datasets switch taxonomic resolution (e.g. from genus to species level) for a given taxon part-way through the time series. This is particularly the case for data from Denmark within the Baetidae, Brachycentridae, Chironomidae, Gammaridae, Oligochaeta and Simuliidae. We did not alter these names because they represent the original information provided for and published in Haase *et al*.^1^, and standardisation methods may vary depending on intended future use. In the time series provided, these issues could affect analyses of shifts in community composition, which could reflect a shift in identification level rather than compositional change. These issues do not affect analyses of total abundance and have little influence taxa richness or diversity, including their temporal trends, as they are typically substitutions of one unique taxon for another. As with all large datasets of ecological time series, data users should carefully check the data considering their intended use and when questions arise, contact data providers (listed in TREAM_siteLevel.csv).

Finally, since each record was assigned the Hydrography90m stream network^25^ subcatchment, users can directly interact with the *hydrographr* R-package which facilitates subsequent network and distance analyses using the data records^32^.

## Data Availability

Code used to conduct analyses for the corresponding publication analysing these data^2^ is available at: https://github.com/Ewelti/EuroAquaticMacroInverts. Code to calculate site and year level biodiversity metrics is available at: https://github.com/Ewelti/EuroAquaticMacroInverts/blob/main/R/Initial_Biodiversity_FuncTrait_and_climate_calcs/TaxaDiversity_calculation.R.
